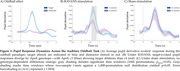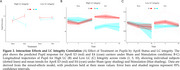# Genotype‐Dependent Modulation of LC‐Mediated Arousal by RAVANS: Evidence from Pupil Responses in an Oddball Task

**DOI:** 10.1002/alz70861_108943

**Published:** 2025-12-23

**Authors:** Gabriel Wainstein, Maxime Van Egroo, Marion Baillet, Joost M. Riphagen, Lukas Heinrich, Jill Grube, Nina Engels, Ronald Garcia, Roberta Sclocco, Vitaly Napadow, Heidi I.L. Jacobs

**Affiliations:** ^1^ Athinoula A. Martinos Center for Biomedical Imaging, Massachusetts General Hospital and Harvard Medical School, Boston, MA USA; ^2^ Spaulding Rehabilitation Hospital, Harvard Medical School, Charlestown, MA USA

## Abstract

**Background:**

Respiratory‐associated vagus nerve stimulation (RAVANS) enhances arousal via ascending neuromodulation. We applied RAVANS during an auditory oddball task, measuring evoked pupil dilations (PD, peak derivative)—a marker of locus coeruleus (LC) phasic activity—to assess its effect on attention. Given LC’s early involvement in Alzheimer’s disease (AD) tau pathology, we examined PD differences by ApoE genotype and MRI‐based LC Integrity to explore AD‐risk vulnerabilities and therapeutic windows.

**Method:**

Cognitively normal older adults (mean age: 67.4, 49% female, 34% ApoE‐E4 carriers) underwent a multi‐visit protocol with blood draws and cross‐over 7T MRI sessions using RAVANS or sham stimulation. Participants completed a two‐week stimulation period (10 visits), with three auditory oddball sessions (Visit 1, 5, 10) for PD measurements. Mixed‐effects linear regression models tested the effects of condition, ApoE, and LC Integrity on PD across visits.

**Result:**

Oddball stimuli increased PD compared to standard stimuli (Figure 1A) with RAVANS boosting PD for E3 vs. E4 between 560–840 ms (Figure 1B; *p* <0.05; 1,000‐iteration non‐parametric permutation of two‐sample t‐tests), but no on sham (Figure 1C; p_perm_>0.05). Furthermore, RAVANS modulated PD with a three‐way interaction between Treatment, ApoE, and LC Integrity (estimate = 0.0909, *p* = 0.002; Figure 2A). Post‐hoc tests showed Stimulation decreased PD for E4 carriers with High LC Integrity (Stimulation vs Sham: estimate = ‐0.0498, *p* = 0.0071), while E3 carriers with High LC showed increased PD (estimate = 0.0363, *p* = 0.0127). No effects were observed for Low LC Integrity (E4: *p* = 0.6360; E3: *p* = 0.9140; Figure 2B‐C). Longitudinal trajectories for High LC Integrity showed sustained PD increases for E3 under Stimulation across visits (Figure 2B‐C).

**Conclusion:**

RAVANS modulates LC‐mediated attention in a genotype‐ and LC Integrity‐dependent manner, with significant effects in ApoE E4 carriers. These findings highlight the potential for personalized neuromodulation strategies to optimize arousal in AD risk management, particularly for E4 carriers with preserved LC Integrity. Future studies will explore additional cognitive and neurobiological factors, such as tau pathology and LC activity, to refine RAVANS applications for early AD intervention.